# Ginsenoside Re Treatment Attenuates Myocardial Hypoxia/Reoxygenation Injury by Inhibiting HIF-1α Ubiquitination

**DOI:** 10.3389/fphar.2020.532041

**Published:** 2020-09-09

**Authors:** Huiyuan Sun, Shukuan Ling, Dingsheng Zhao, Jianwei Li, Yang Li, Hua Qu, Ruikai Du, Ying Zhang, Feng Xu, Yuheng Li, Caizhi Liu, Guohui Zhong, Shuai Liang, Zizhong Liu, Xingcheng Gao, Xiaoyan Jin, Yingxian Li, Dazhuo Shi

**Affiliations:** ^1^State Key Laboratory of Space Medicine Fundamentals and Application, China Astronaut Research and Training Center, Beijing, China; ^2^Science and Technology Department, Beijing University of Chinese Medicine Third Affiliated Hospital, Beijing, China; ^3^National Clinical Research Center for Chinese Medicine Cardiology, Xiyuan Hospital, China Academy of Chinese Medical Sciences, Beijing, China; ^4^State Key Laboratory of Proteomics, Beijing Proteome Research Center, National Center for Protein Sciences Beijing, Beijing Institute of Lifeomics, Beijing, China

**Keywords:** ginsenoside Re, cardiomyocytes, hypoxia/reoxygenation injury, HIF-1α, ubiquitination

## Abstract

Previous studies have shown an attenuating effect of ginsenoside Re on myocardial injury induced by hypoxia/reoxygenation (H/R). However, the underlying mechanism remains unclear. This study was designed to determine the underlying mechanism by which ginsenoside Re protects from myocardial injury induced by H/R. HL-1 cells derived from AT-1 mouse atrial cardiomyocyte tumor line were divided into control, H/R, and H/R + ginsenoside Re groups. Cell viability was measured by CCK-8 assay. ATP levels were quantified by enzymatic assays. Signaling pathway was predicted by network pharmacology analyses and verified by luciferase assay and gene-silencing experiment. The relationship between ginsenoside Re and its target genes and proteins was analyzed by docking experiments, allosteric site analysis, real-time PCR, and ubiquitination and immunoprecipitation assays. Our results showed that ginsenoside Re treatment consistently increased HL-1 cell viability and significantly up-regulated ATP levels after H/R-induced injury. Network pharmacology analysis suggested that the effect of ginsenoside Re was associated with the regulation of the Hypoxia-inducing factor 1 (HIF-1) signaling pathway. Silencing of *HIF-1α* abrogated the effect of ginsenoside Re on HL-1 cell viability, which was restored by transfection with an HIF-1α-expressing plasmid. Results of the bioinformatics analysis suggested that ginsenoside Re docked at the binding interface between HIF-1α and the von Hippel-Lindau (VHL) E3 ubiquitin ligase, preventing VHL from binding HIF-1α, thereby inhibiting the ubiquitination of HIF-1α. To validate the results of the bioinformatics analysis, real-time PCR, ubiquitination and immunoprecipitation assays were performed. Compared with the mRNA expression levels of the H/R group, ginsenoside Re did not change expression of *HIF-1α* mRNA, while protein level of HIF-1α increased and that of HIF-1α[Ub]n decreased following ginsenoside Re treatment. Immunoprecipitation results showed that the amount of HIF-1α bound to VHL substantially decreased following ginsenoside Re treatment. In addition, ginsenoside Re treatment increased the expression of GLUT1 (glucose transporter 1) and REDD1 (regulated in development and DNA damage response 1), which are targets of HIF-1α and are critical for cell metabolism and viability. These results suggested that Ginsenoside Re treatment attenuated the myocardial injury induced by H/R, and the possible mechanism was associated with the inhibition of HIF-1α ubiquitination.

## Introduction

Although coronary blood flow restoration rescues the ischemic myocardium, myocardial ischemia/reperfusion (I/R) may lead to sustained and even irreversible myocardial ultrastructural damage (Chen et al., 2019). Myocardial I/R injury, reported to be a complicated process involving many signaling pathways, results in apoptosis and necrosis of cardiomyocytes and counteracts the effect of reperfusion therapy (Li et al., 2019). At supra-cellular level, even dysfunctional changes in heart muscle without necrosis, such as fatal arrhythmia and cardiogenic shock, may create a danger to the heart (Refaey et al., 2019).

A previous study demonstrated that ginsenoside Re, a component of Panax ginseng, protects cardiomyocytes against H/R injury (Lim et al., 2013); however, the mechanism remains unclear. This study was designed to test whether the underlying mechanism of ginsenoside Re in attenuating myocardial H/R injury was associated with inhibiting HIF-1α ubiquitination.

## Material and Methods

### Materials

Ginsenoside Re with a purity of 92.3% was purchased from the National Institute of Food and Drug Control (No. 110754-201525). HL-1 cells were gifted by Professor Yanzhong Chang (Hebei Normal University). The hypoxia response element (HRE)-luc plasmid was gifted by Professor Tatsuya Kobayashi (Endocrine Unit, Massachusetts General Hospital, Harvard Medical School). The HA-HIF-1α plasmid was purchased from Addgene. The His-ubiquitin plasmid was gifted by Professor Lingqiang Zhang (State Key Laboratory of Proteomics, Beijing Proteome Research Center, National Center for Protein Sciences Beijing, Beijing Institute of Lifeomics). Anti-Myc and anti-Flag monoclonal antibodies were purchased from Cell Signaling (Danvers, Massachusetts, USA) and Sigma (St Louis, Missouri, USA), respectively. SiRNAs targeting the HIF-1α gene were designed and synthesized by Shanghai GenePharma Co., Ltd.

The HIF-1α siRNA sequence was 5′-UAAUAUCUUCUUUAUUGUCCU-3′.

### Cell Culture, Model, and Treatment

HL-1 cells, from a cell line derived from the AT-1 mouse atrial cardiomyocyte tumor lineage, retain the *in vitro* phenotypic characteristics of adult cardiomyocytes (Vicencio et al., 2015) and were cultured with supplemented Claycomb medium comprising 90% Claycomb medium (JRH Biosciences, 51800C-500 ML), 10% fetal bovine serum (Sigma-Aldrich, F2442-500 ML), 100 U/ml:100 μg/ml penicillin/streptomycin (Life Technologies, 15140-122), 0.1 mM norepinephrine (Sigma, A-0937; 10 mM stock), and 2 mM L-glutamine (Life Technologies, 25030-081; 200 mM stock). The HL-1 cells were passaged every two or three days.

The HL-1 cells were divided into control, H/R, and H/R + Re groups. The cells in the control group were maintained under normoxic conditions (21% O_2_, 5% CO_2_, and 74% N_2_) for 19.5 h. The HL-1 cells in the H/R group were cultured in a hypoxic and sugar-free chamber containing 95% N_2_ and 5% CO_2_ for 18 h and then placed in a normal chamber containing 21% O_2_, 5% CO_2_, and 74% N_2_ for 1.5 h (Vicencio et al., 2015). The HL-1 cells in the H/R + Re group were treated with 100 µM ginsenoside Re in the and otherwise treated the same as the cells in the H/R group.

### Cell Viability

Cell viability was measured with Cell Counting Kit-8 (CCK-8) (CK04; Dojindo, Japan) according to the manufacturer’s instructions. Briefly, 10 µl of CCK-8 solution was added to each well (100 µl medium) and incubated for 1 h at 37°C, and then, the absorbance was measured at 450 nm in a microplate reader (BL941; Berthold, Germany).

### ATP Level

The ATP levels were quantified using a commercial kit (A095-1; Nanjingjiancheng, Nanjing, China) according to the manufacturer’s instructions.

### Network Pharmacology Prediction

The drug targets predicted by BATMAN-TCM were filtered using a Perl script with a confidence score cut-off of 3.0. These selected drug targets were mapped to human and rat orthologs. Pairwise ortholog mapping files (“human to rat”) were downloaded from the InParanoid database (Release 8.0) (Sonnhammer and Östlund, 2014). The statistics from the KEGG pathway enrichment analysis of the selected drug targets were generated using the clusterProfiler package in R.

### Docking Analysis

The Schördinger Maestro package was used to perform a dock analysis. The 2D ligand structure of the ginsenoside Re molecule was downloaded from PubChem (https://pubchem.ncbi.nlm.nih.gov)and prepared using LigPrep. VHL and HIF-1α protein structure was modeled with I-TASSER and constructed following the Protein Prepare Wizard workflow in the Maestro package (Roy et al., 2010). All chains of the structure were used to prepare the receptor, and all water molecules greater than 5 angstroms around the protein were removed. The potential allosteric sites of VHL and HIF-1α were predicted by AllositePro (Song et al., 2017). The prepared ligand was then flexibly docked into the predicted binding site of the receptor using the Glide XP mode with default parameters. Finally, several docking poses were obtained for the molecule, and the one with the best Glide score was chosen: The Glide score of ginsenoside Re docking with the VHL protein was -7.936, and the Glide score of ginsenoside Re docking with the HIF-1α protein was -7.391.

### Allosteric Site Prediction

AllositePro is used to predict allosteric sites in proteins by combining pocket features with outcomes of a perturbation analysis. This feature-based model was trained with a high-quality benchmarking data set, ASBench (Huang et al., 2015), on the basis of a logistic regression method. Then, normal-mode analysis (NMA), an efficient means to study likely motion of interacting biomolecules, was used to evaluate the dynamic changes in proteins triggered by allosteric ligands. The score of the perturbation method (PNMA) was defined by the p-value determined by a Wilcoxon–Mann–Whitney test. In the final model, an allosteric site was identified by combining AllositePro and NMA data.

### Luciferase Reporter Assay

HL-1 cells were seeded on 24-well plates (5 × 10^4^ cells per well) and transfected with pRL-TK and HRE-luc reporter gene plasmids with or without ginsenoside Re (100 µM) treatment. Luciferase activity was measured as described (Ling et al., 2012; Clément et al., 2015). To normalize the results, Renilla luciferase was cotransfected. Luciferase activity was detected by a Dual-Luciferase^®^ reporter assay system (Promega).

### Real-Time PCR

Total RNA was extracted from the HL-1 cells using TRIzol reagent (TaKaRa, Kyoto, Japan) according to the manufacturer’s instructions. Real-time PCR was performed in duplicate on a Light Cycler (Eppendorf, Hamburg, Germany) using SYBR green mix (S4438; Bio-Rad, Hercules, CA), and the expression of glucose transporter 1 (GLUT1) and regulated in development and DNA damage response 1 (REDD1) was normalized to that of GAPDH. The following primers were used:

GLUT1 sense primer: 5′-GTAGAGCGAGCTGGACGACG-3′;

GLUT1 antisense primer: 5′-GGCCACGATGCTCAGATAGG-3′;

REDD1 sense primer: 5′-CTTCGTCCTCGTCTCGAACT-3′;

REDD1 antisense primer: 5′-CCATCCAGGTATGAGGAGTCTTCC-3′;

GAPDH sense primer: 5′-ACTCCACTCACGGCAAATTCA-3′; and

GAPDH antisense primer: 5′-GGCCTCACCCCATTTGATG-3′.

### Half-Life of HIF-1α Protein Degradation

HL-1 cells were seeded on 24-well plates and transfected with the HA-HIF-1α plasmid (100 ng/well). After culturing for 8 h, the cells were treated with ginsenoside Re (100 µM) or DMSO. Once the cells reached 70% confluence on the plates, cycloheximide (diluted 1:10,000) was added to the plates. Then, the cells were harvested every 2 h until 10 h after cycloheximide treatment. The level of HIF-1α protein was detected by Western blotting and quantified by ImageJ software. The HIF-1α protein half-life was calculated by linear regression analysis.

### Immunoprecipitation and Immunoblotting

Lipofectamine 2000 (Invitrogen, USA) was used for transfection. Forty-eight hours after transfection, the HL-1 cells were harvested and lysed in 1 mL of lysis buffer (1% NP40, 10% glycerol, 135 mM NaCl, 20 mM Tris, 40 µl of 50× cocktail, 40 µl of 50×Na_3_VO_4_, 40 µl of 50×NaF, 40 µl of 50×Na_2_PO_4_, and 0.8 Ml of sterile water, pH 7.4). To determine the interaction between VHL and HIF-1α, immunoprecipitation was performed with 2 µg of agarose-conjugated rabbit anti-HIF-1α antibody (Cell Signaling). The proteins in both the total cell lysates and the immunoprecipitates were measured by immunoblotting in the presence of antibodies against VHL (diluted 1:1,000) and HIF-1α (diluted 1:1,000) (Ling et al., 2012).

### Ubiquitination Assay

To detect exogenously induced ubiquitylation of HIF-1α, the HL-1 cells were transfected with His-Ub and HA-HIF-1α. The cells were treated with or without ginsenoside Re (100 µM), and then, the proteasome inhibitor MG132 (20 µM; Sigma) was added to the medium for 6 h before cell harvesting. Cell lysates were immunoprecipitated with the HA antibody (2 µg, #3724S, Cell Signaling Technology), followed by immunoblotting with anti-His antibody (diluted 1:1000, PM020-8, MBL).

### Statistical Analyses

The data, represented as the mean ± standard error of the mean (SEM), were analyzed by one-way ANOVA. Post hoc analyses included the Student–Newman–Keuls method and Dunnett’s test. Probability values <0.05 were considered significant. We used two-way analysis of variance with unequal variances to account for 2 factors and their interactions. Bonferroni’s adjustment was used for multiple comparisons. Statistical analyses were performed using GraphPad Prism 5 software (GraphPad Software Inc., San Diego, CA).

## Results

### Ginsenoside Re Increased Cell Viability and ATP Levels in HL-1 Cells After H/R Treatment

**Figure 1:**
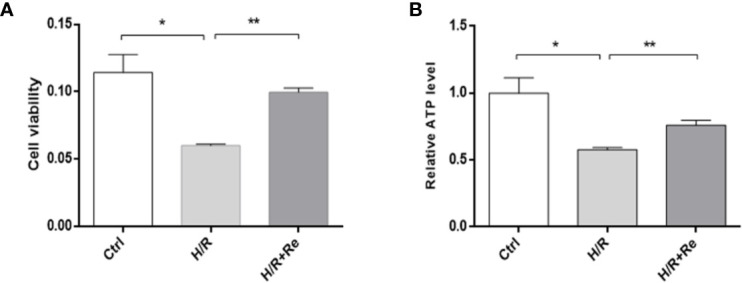
HL-1 cells viability and ATP level after H/R. **(A)** HL-1 cells viability (n=5). **(B)** ATP levels (n = 5). All data are shown as mean ± SEM, *P < 0.05, **P < 0.01. Ctrl, control; H/R, hypoxia reoxygenation; RE, ginsenoside Re; ATP, adenosine triphosphate.

Compared with the control group, H/R treatment reduced HL-1 cell viability, while ginsenoside Re treatment increased cell viability following H/R injury (**Figure 1A**). To validate the role of ginsenoside Re treatment on the energy metabolism of the cardiomyocytes, ATP levels were measured by enzymatic assays. A significant decrease in ATP level was observed after H/R induction, but the ATP level increased after ginsenoside Re treatment (**Figure 1B**).

### Ginsenoside Re Treatment Attenuated Myocardial H/R Injury *via* the HIF-1 Signaling Pathway

**Figure 2:**
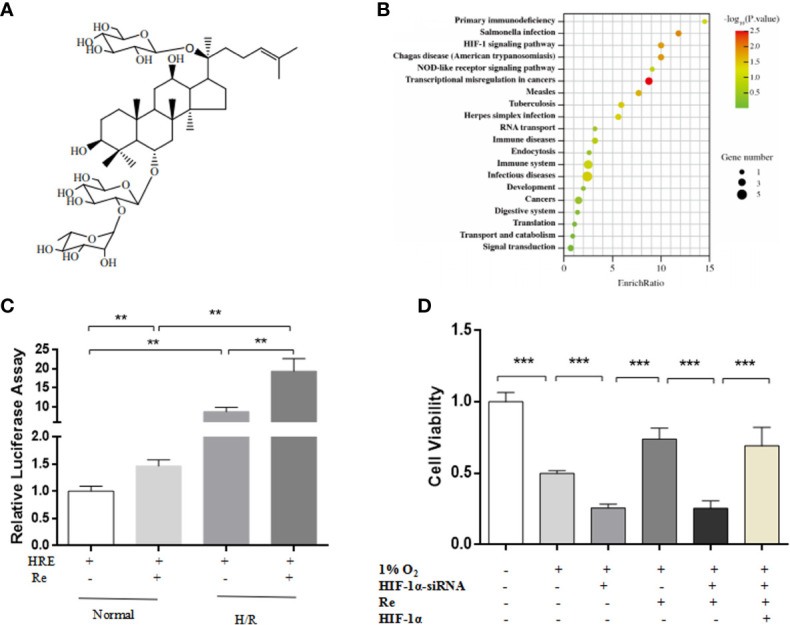
HIF Signaling pathway was predicted by notework pharmacology and vadaliated by luciferase assay and gene siliencing. **(A)** Molecule structure of ginsenoside Re. **(B)** Signaling pathway predictd by network pharmacology. **(C)** Expression of HIF-1α signaling pathway was examined by relative luciferase assay (n = 5). **(D)** HL-1 cell viability after silencing HIF-1α (n = 5). All data are shown as mean ± SEM, **P < 0.01, ***P < 0.001. H/R, hypoxia reoxygenation; RE, ginsenoside Re.

A network pharmacology prediction analysis was performed to explore the potential molecular mechanism of ginsenoside Re action. The HIF-1 signaling pathway was predicted to be one of the most important pathways involved in the regulation of H/R (**Figures 2A, B**). To validate the signaling pathway predicted by the network pharmacology analysis, a luciferase reporter assay was performed. Compared with the HRE activity of the H/R group, treatment with ginsenoside Re increased the activity of HRE (**Figure 2C**). Inhibiting the HIF-1 signaling pathway by silencing *HIF-1α* attenuated the effect of ginsenoside Re on HL-1 cell viability, and the effect was reestablished after transfection with the HIF-1α-expressing plasmid (**Figure 2D**).

### Ginsenoside Re Treatment Inhibits the Ubiquitination of HIF-1α

**Figure 3:**
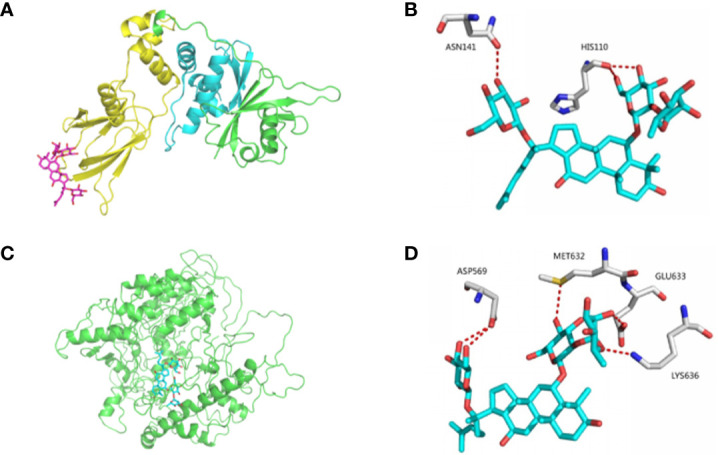
The bindings among ginsenoside Re, VHL protein and HIF-1α protein. **(A, B)** The details of predicted binding mode of ginsenoside Re and VHL protein. The contact residues were shown and labeled by type and number. The red dotted line illustrated the hydrogen bond interaction. **(C, D)** The details of predicted binding mode of ginsenoside Re and HIF-1α protein. The contact residues were shown and labeled by type and number. The red dotted line illustrated the hydrogen bond interaction.

Docking and allosteric site analyses were performed to analyze the binding site where ginsenoside Re interacts with HIF-1 to initiate the signalling pathway, and the results showed that ginsenoside Re was incorporated into the binding interface of HIF-1α and the VHL protein, preventing VHL from binding HIF-1α and inhibiting the ubiquitination of HIF-1α (**Figures 3A–D**).

**Figure 4:**
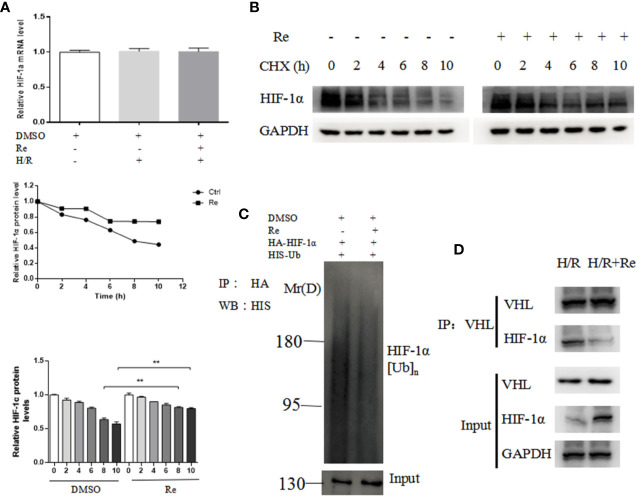
Ubiquitination of HIF-1α following ginsenoside Re treatment. **(A)** HIF-1α mRNA level. **(B)** Half-life of HIF-1α protein. **(C)** HIF-1α ubiquitination following ginsenoside Re treatment. **(D)** Interaction between VHL and HIF-1α following ginsenoside Re treatment. All data are shown as mean ± SEM, **P < 0.01. Ctrl, control; RE, ginsenoside Re; DMSO, Dimethyl sulfoxide.

To validate the results of the bioinformatic analysis, real-time PCR and ubiquitination and immunoprecipitation assays were performed. Compared with the HIF-1α mRNA level in the H/R group, ginsenoside Re treatment did not change the level of HIF-1α mRNA (**Figure 4A**); however, following ginsenoside Re treatment, the level of HIF-1α increased (**Figure 4B**) and that of HIF-1α[Ub]n decreased (**Figure 4C**). The immunoprecipitation results showed that the quantity of HIF-1α binding to VHL decreased following ginsenoside Re treatment (**Figure 4D**).

### Ginsenoside Re Treatment Increases the Expression of HIF-1α Target Genes

**Figure 5:**
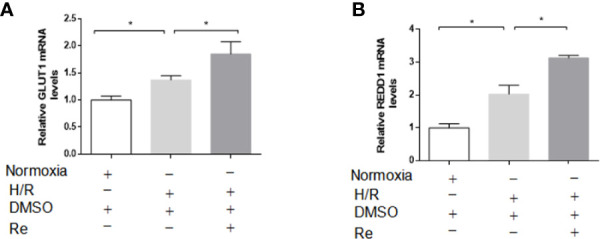
Effects of ginsenoside Re on gene targets of HIF-1α. **(A, B)** mRNA levels of GLUT and REDD1 were measured by qPCR (n=5). All data are shown as mean ± SEM, *P < 0.05. H/R, hypoxia reoxygenation; RE, ginsenoside Re; DMSO, Dimethyl sulfoxide.

GLUT1 and REDD1 are the target genes of HIF-1α, and they are associated with energy metabolism and cell apoptosis, respectively. Real-time PCR was performed to determine the expression of the GLUT1 and REDD1 mRNA levels. Compared with their expression in the H/R group, ginsenoside Re treatment increased the expression of GLUT1 and REDD1 (**Figures 5A, B**).

## Discussion

In the present study, we demonstrated that ginsenoside Re treatment improved HL-1 cell viability and ATP levels following H/R. In addition, inhibiting the HIF-1 signaling pathway by silencing *HIF-1α* counteracted the effect of ginsenoside Re on HL-1 cell viability. Furthermore, the results of the bioinformatics analysis demonstrated that ginsenoside Re acted upon the binding interface between HIF-1α and VHL protein, preventing VHL from binding HIF-1α, thus inhibiting the ubiquitination of HIF-1α. To validate the results of the bioinformatic analysis, real-time PCR and ubiquitination and immunoprecipitation assays were performed. Compared with the level of HIF-1α mRNA in the the H/R group, ginsenoside Re treatment did not change the level of HIF-1α mRNA; however, following ginsenoside Re treatment, the level of HIF-1α increased and that of HIF-1α[Ub]n decreased. The results from the immunoprecipitation showed that the quantity of HIF-1α binding to VHL decreased following ginsenoside Re treatment.

HIF-1, composed of the HIF-1α functional subunit and the HIF-1β constitutional subunit (Luo et al., 2019), plays an important role in sensing and adapting to changes in oxygen concentration and is associated with cellular energy metabolism and apoptosis (Soni and Padwad, 2017). HIF-1 is an important target for diseases related to oxygen deprivation (Albadari et al., 2019). Some HIF-1α-based examples including antioxidation (iNOS), erythropoiesis (EPO) and angiogenesis (VEGF, ENG, LEP), counteract ischemia, hypoxia and promote angiogenesis (Jain et al., 2018). Previous studies demonstrated that HIF-1 activation and upregulation of HIF-1α attenuate myocardial I/R injury (Yang et al., 2020). VHL, a tumor suppressor, functions as part of a ubiquitin ligase complex that recognizes HIF-1α as a substrate and is part of the oxygen-sensing mechanism of the cell (Vriend and Reiter, 2016). Under normoxic conditions, α-ketoglutarate-dependent prolyl hydroxylases (PHDs) catalyze the hydroxylation of proline residues in the oxygen-dependent degradation domains of HIF-1α, which are recognized by the VHL E3 ubiquitin ligase complex, leading to HIF-1α ubiquitination and subsequent degradation (LaGory and Giaccia, 2016). Inhibiting the combination of VHL and HIF-1α is an important step to decrease the ubiquitination of HIF-1α and improve myocardial cell viability. However, to date, no effective drugs have been developed for inhibiting the ubiquitination of HIF-1α (Zeng et al., 2017). The present study shows that ginsenoside Re treatment has a protective effect against myocardial injury induced by H/R through the HIF-1 signaling pathway, and the results show that ginsenoside Re inhibited the combination of HIF-1α and VHL protein, thus inhibiting the ubiquitination of HIF-1α. ODDD domain in HIF-1α mediates its binding with VHL (Warren et al., 2019). It is important to determinate the specific site in this domain for ginsenoside Re binding.

Glucose transporters (Gluts) are involved in the transmembrane facilitated diffusion of glucose (Lu et al., 2019). Among them, GLUT1 is a basal glucose transporter that is widely expressed in cells and tissues (Mueckler and Thorens, 2013). In particular, GLUT1 is critical for the constant transport of glucose into erythrocytes through facilitated diffusion (Nagao et al., 2019). In our study, ginsenoside Re treatment increased the mRNA level of GLUT1, suggesting that ginsenoside Re improves energy metabolism in cardiomyocytes following H/R. REDD1 is an essential regulator of cell growth, and a low level of REDD1 expression is associated with cell apoptosis (Sun and Yue, 2019). Our study showed that ginsenoside Re treatment significantly increased the mRNA level of REDD1, indicating that the effects of ginsenoside Re on cardiomyocyte H/R injury are related to the inhibition of cell apoptosis.

GLUT1 is related to glucose transport, which is a part of energy metabolism. AMPK is a regulator of energy metabolism in the heart and is activated by falling energy status that boosts ATP production by stimulating glucose uptake and glycolysis (Cisternas et al., 2019). In our study, ginsenoside Re treatment increased the level of ATP and the mRNA level of GLUT1 following H/R, which implicated that the AMPK signaling pathway was involved in the regulation of ginsenoside Re on H/R. REDD1 regulates mTOR-dependent phosphorylation following AMPK activation, energy stress induces expression of REDD1, inhibiting mTOR activity, inhibits protein synthesis and decreases cellular growth (Seong et al., 2019). In the present study, ginsenoside Re treatment significantly increased the mRNA level of REDD1, which indicates that the effects of ginsenoside Re on cardiomyocytes H/R injury are mTOR signaling pathway-related. BNIP3, which is a pro-apoptotic protein and a member of Bcl-2 family, is the downstream of HIF-1 signal pathway (Cai et al., 2017). HIF-1α/BNIP3 signaling pathway-induced-autophagy plays protective role during myocardial ischemia-reperfusion injury (Zhu et al., 2020). HIF-1α/BNIP3 signaling pathway will be explored in our next study. In the future, the underlying mechanisms will be further explored. The changes of AMPK/mTOR/HIF-1/BNIP3 signal pathways will be further clarified and the specific sites for Re binding domain will be identified in the next experiment.

**Figure 6:**
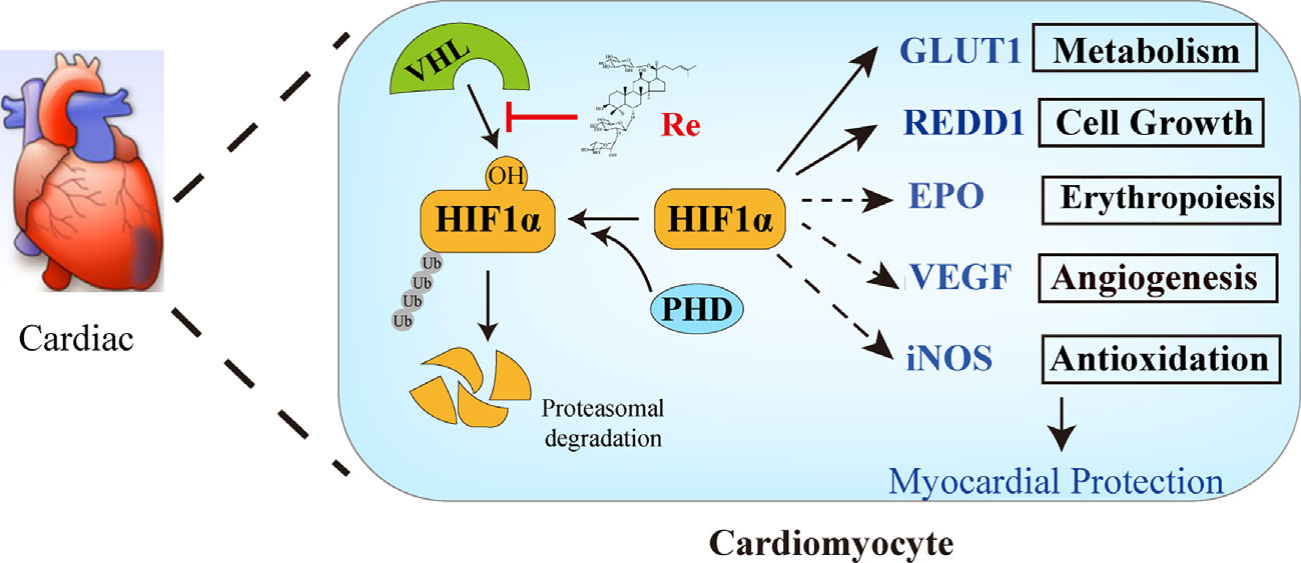
The possible mechanism of ginsenoside Re attenuating myocardial injury induced by H/R. Ginsenoside Re inhibited the ubiquitination of HIF-1α *via* inhibiting the binding between VHL and HIF-1α. In addition, the target genes of HIF-1α, such as GLUT1 and REDD1, increased following ginsenoside Re treatment.

Taken together, our present work has demonstrated that ginsenoside Re attenuated the myocardial injury induced by H/R. The possible mechanism is associated with the localization of ginsenoside Re at the binding interface between HIF-1α and VHL protein, preventing VHL from binding HIF-1α, thereby inhibiting the ubiquitination of HIF-1α (**Figures 6**).

## Data Availability Statement

The datasets analyzed in this article are not publicly available because further study will be done based on parts of these datasets as we mentioned in this paper. Requests to access the datasets should be directed to C729@bucm.edu.cn.

## Author Contributions

DS and YXL conceived the study. HS performed the experiment with support from SKL, DZ, JL, YHL, and CL. YL performed network pharmacology analysis. RD and GZ analyzed and interpreted the results. SL, ZL, XG, XJ, and FX provided intellectual contribution. HS wrote the manuscript under the guidance of DS, YXL, and SKL. DS, YXL, SKL, HQ, and YZ revised the manuscript and gave final approval of the submitted manuscript. All authors contributed to the article and approved the submitted version.

## Conflict of Interest

The authors declare that the research was conducted in the absence of any commercial or financial relationships that could be construed as a potential conflict of interest.
